# The Relationship between Foot Status and Motor Status in Preschool Children: A Simple, Comparative Observational Study

**DOI:** 10.3390/healthcare9080936

**Published:** 2021-07-26

**Authors:** Milan Kojić, Branka Protić Gava, Milan Bajin, Marko Vasiljević, Jasmina Bašić, Dušan Stojaković, Milena P. Ilić

**Affiliations:** 1 Faculty of Sport and Physical Education, University of Novi Sad, 21000 Novi Sad, Serbia; pokretzaokret@yahoo.com (M.K.); brankapg@gmail.com (B.P.G.); marko.vasiljevic.rzsport@gmail.com (M.V.); 2 Faculty of Sport and Tourism, Educons University in Novi Sad, 21000 Novi Sad, Serbia; milanbajin@yahoo.com; 3 The College of Health Sciences, Academy of Applied Studies Belgrade, 11000 Belgrade, Serbia; basic.jasmina23@gmail.com; 4 Faculty of Contemporary Arts, University Business Academy in Novi Sad, 11000 Belgrade, Serbia; dusan.stojakovic@fsu.edu.rs

**Keywords:** preschool age, flat feet, baropodometry, motor skills

## Abstract

Background: The research objective of the study is to determine the differences in the manifestation of the motor status of normally fed preschool test subjects, classified into groups according to foot status. Methods: This is a simple, comparative observational study. Preschool children included in this study have been subjected to anthropometric measurements in order to determine BMI, tests for motor skills assessment (running at 20 m from a high start, standing broad jump, backwards polygon, rectangular seated forward bend, plate tapping, sit-ups for 60 s, and bent arm hang), and a determination of foot status. The total sample was comprised of 202 test subjects who attended a regular sports program, aged 3.9 to 6.5 years of decimal age (M = 141; Age = 5.3 ± 0.74; Height = 117.3 ± 7.1; Weight = 22 ± 3.7; F = 61; Age = 5.1 ± 0.73; Height = 114.9 ± 7.4; Weight = 21.2 ± 3.8), of which 153 (75.7%) were normally fed, 6 (3%) were undernourished, 30 were overweight (14.9%), and 13 were obese (6.4%). Results: In the total sample, 30 (14.9%) subjects had normal arch feet, 90 (44.6%) high arched feet, and 41 (20.3%) flat feet. We found 41 (20.3%) subjects who had different left and right foot statuses within this sample. The data were processed by means of nonparametric tests (the Kruskal–Wallis and Mann–Whitney U tests) at a significance level *p* ≤ 0.05. Conclusion: The results show that there is a statistically significant difference between groups of subjects with different foot statuses in the manifestation of motor status in most tests, with a significance level of *p* ≤ 0.01, and in tests of sit-ups for 60 s and the bent arm hang, there is a statistically significant difference, the level of which is *p* ≤ 0.05. It is only in the inclination test of rectangular seated forward bend that no statistically significant difference was displayed.

## 1. Introduction

Foot posture represents a key factor both in standing and walking [1,2]. In the standing position in children and adults, a healthy foot rests on three points: the heel bone, the joint of the first metatarsal, and the fifth metatarsal bone. The arches extending between these three points form the structures that constitute the longitudinal and transverse arches of the feet. The longitudinal arches of the feet are the longitudinal medial and lateral arches, and the transverse ones are the posterior and anterior (metatarsal) transverse arches [3]. These structures are flexible and adaptable in every situation, and the tendons and joints between the bones have an attenuating effect in motion. The modern lifestyle has significantly changed the original purpose of the human foot. All healthy babies are born with flexible flat feet. The medial longitudinal arch of their feet develops during the first ten years of life [4]. The progression of medial longitudinal arch elevation continues with increasing age [5].

The foot status of preschool children represents a great controversy. Parents often become worried due to this issue, and paediatricians often prescribe suspicious and unnecessary treatments without adequate diagnostic analysis [6]. The condition of the foot status of children of this age causes controversy, primarily relating to the definition of a flat foot. Due to variable patterns and age groups, the paediatric flat foot varies in the literature by 3–15% [7]. For example, a study of 835 children found that the longitudinal arch of the foot was reduced from 54% in three-year-olds to 24% in six-year-olds [6]. This result coincides with findings in other studies, in which it is ascertained that there is a continuous decrease in foot drop between 4 and 10 years of age [8,9]. Additional confusion is brought about due to various diagnostic procedures, which define feet status: somatotopic methods in statics and dynamics, scanner-generated footprints, footprints obtained by ink printing, radiography, clinical tests, and baropodometry in statics and dynamics [10,11,12].

Preschool age represents a very sensitive developmental age. The childhood period is essential for acquiring and developing motor skills. Preschool age (from 4 to 7 years) is the period when the structure of the motor space is built based on genetic and external factors that affect the overall growth and development of children [13]. The motor functioning of preschool children is of a general type, meaning there are no differentiated motor abilities yet because children react with the whole body and overall motor skills [14,15]. The feature of this age is very distinct developmental integrity. A tight connection between childhood developmental domains has been noted, as is the case with physical, motor, cognitive domains, etc. The development of one domain influences the development of other domains; motor skills play a vital role because they initiate numerous developmental stimuli, which favourably affect the child’s development in its entirety. Early experiences of movement, teaching, adequate space and positive attitude of parents, educators, etc., enable the optimal motor development of children [16].

The analysis of the relationship between the foot status and motor abilities of children of this age remains an insufficiently investigated field in scientific papers. Therefore, our goal is to determine whether children with normal nutrition with differing feet statuses show different results in motor tests that are statistically significant from subjects with normal and high arched feet.

## 2. Materials and Methods

This is a simple comparative observational study. Preschool children included in this study have been subjected to anthropometric measurements to determine their BMI, tests for motor skills assessment, and a determination of their foot status. The test subjects in this research are participants of both sexes from the program of the chosen sports school, of preschool age ranging from 3.9 to 6.5 years of decimal age (5.3 years ± 7.4 SD). The criterion for inclusion in the study was that the children were healthy and free from neurological disorders.

Only children with normal nutrition were included in the study and were subsequently divided into three groups according to their foot status. The test subjects and their parents were fully informed about the nature and purpose of this study, after which parents gave their consent and signed written consent for their children’s participation in this study. The study was conducted following the ethical standards of the Declaration of Helsinki. We have an Institutional Review Board approval document given by the Ethical Board of the Faculty of Contemporary Arts Belgrade, no. 46/21, dated 5 March 2021.

The paper hypothesises that preschool children with normal feet arches and high arched feet will show better motor status than children with flat feet.

### 2.1. The Sample of Participants

The study included 202 preschool children between 3.9 and 6.5 years of decimal age (M = 141; F = 61). In order to be included in the study, the participants could not be diagnosed with chronic musculoskeletal problems, taking medication, or affected by immobilisation or injury of any part of the body in the six months before the measurement. All participants had attended the sports school program for at least six months, two to three times a week. Test subjects were classified into four groups according to nutritional status: 6 of them were undernourished (3%), 30 overfed (14.9%), 13 obese (6.4%), and 153 normal (75.7%). A total of 49 (24.3%) undernourished, overfed, and obese children were excluded from further research to eliminate the most significant interference factor for the manifestation of motor abilities and the formation of arches, i.e., the current feet status. Out of the 153 normally fed, 21 (13.7%) of the test subjects had a normal foot arch following their age, 70 (45.8%) had high arched feet, 29 (19%) had flat feet, and 33 (21.6%) had a different status for their left and right feet. The subjects with different left and right feet statuses were also excluded from the study to obtain the most detailed results of statistical procedures. Thus, the final sample of test subjects in this study comprises 120 children, 86 males and 34 females, divided into three groups according to the feet status. 

### 2.2. The Direction and Procedures of Measurement

The data utilised in this study were collected within the regular curriculum of the school of sports during the school year 2012/2013 while carrying out initial tests for motor ability and measurement and defining physical status in the up-to-date equipped sports laboratory of the Centre for Sports Orientation, “The Movement for a Turn”. Testing motor ability was carried out during regular classes in the course of the last week of September, and measurements and determining the physical status were carried out in the second half of September and during October.

All instruments, devices, and systems in the sports laboratory had previously undergone strict calibration procedures and the subsequent preparation for laboratory measurements. The measurement procedure was carried out according to a defined protocol for 30 min per subject in the presence of at least one parent. The measuring agents for assessing motor skills tests were selected graduate professors of sports and physical education who had been doing only one individual test for years. Assistants and scorers were undergraduate students. Measurements of postural and morphological status were performed by two graduated kinesitherapists, who are certified scorers for each system and device separately in the implemented protocol [17].

### 2.3. Variables

The implemented protocol of the paper comprised the results of anthropometric measurements utilised to assess nutrition and classification of subjects based on the BMI% percentile, given that the final sample of subjects consisted only of a group including normally fed.

**Figure 1:**
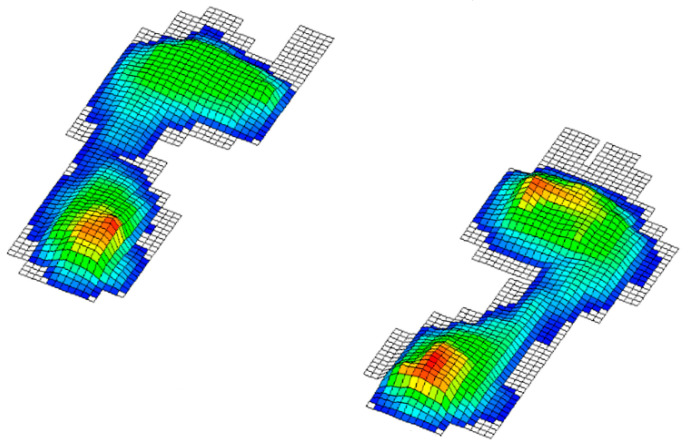
Normal arch feet.

**Figure 2:**
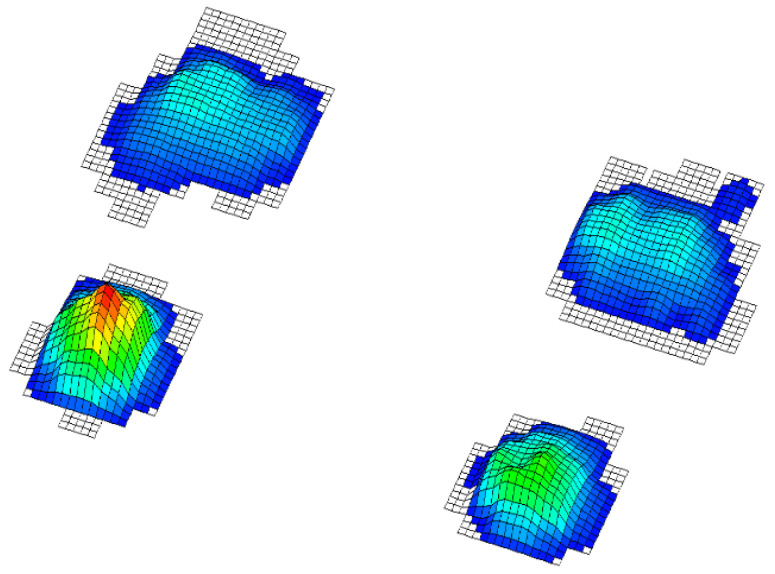
High arched feet.

**Figure 3:**
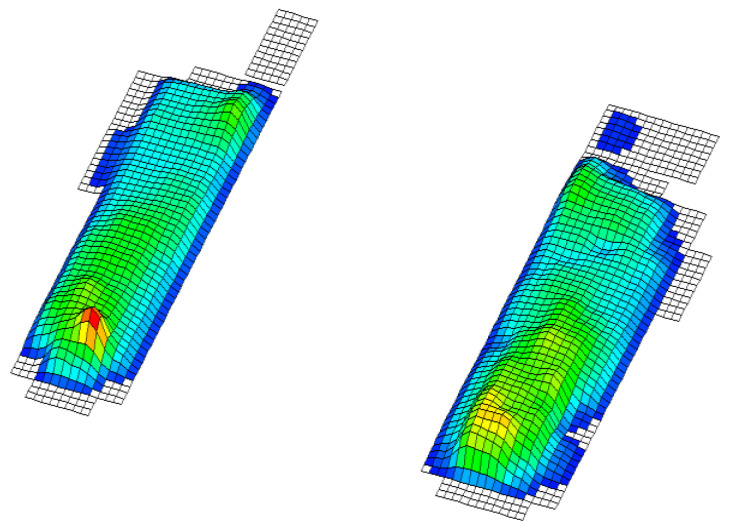
Flat feet.

**Figure 4:**
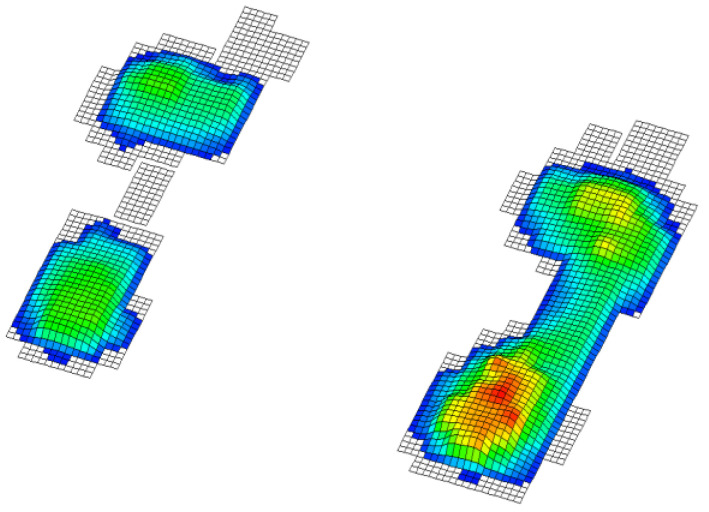
Different left and right feet status.

In this paper, we test the significance of differences between the groups so that a division of variables into two groups has been considered. The independent ones consisted of the results of the applied battery of motor tests and the dependent variables that represent the status of the foot obtained by footprint during baropodometric measurements in standing position; more precisely: 1. normal feet arch (Figure 1), 2. high arched feet (Figure 2), and 3. flat feet (Figure 3) [18]. A group of subjects with different left foot and right foot statuses was excluded from the final sample (Figure 4) [19].

Legend (Figure 1, Figure 2, Figure 3 and Figure 4): Red represents pressures greater than 30 kpa, yellow over 20 kpa, green over 10 kpa, and blue up to 10 kpa. 

#### 2.3.1. Anthropometric Measurements

The following anthropometric measures were taken following the standards and procedures established by the International Biological Program [20]: 1. Body height (TV) was measured with an anthropometer from the company “Seca”, with a measurement accuracy of 0.1 cm; 2. Body mass (TM) was measured with a digital decimal scale from the company “Seca”, with an accuracy of 0.1 kg.

#### 2.3.2. Body Mass Index

The assessment of nutritional status was obtained based on the value of the Body Mass Index (BMI). The appropriate percentiles for age and gender were determined based on reference values and criteria proposed by the World Health Organization [17]. According to the reference values, the subjects were divided into four groups according to nutritional status:

- Malnourished up to the 5th percentile;
- Normally fed from the 6th to the 84th percentile;
- Overweight from the 85th to 94th percentile;
- Obese children beyond the 95th percentile.

#### 2.3.3. Feet Posture

The definition of feet status was performed based on footprint baropodometric measurements in standing position and the classification of groups according to feet status, which comprised:

- Normal arch feet (in conformity with age, given that these are children up to 6.5 years of age);
- High arched feet (without contact of the middle part of the sole, i.e., only with the contact surfaces on the heel and front of the sole);
- Flat feet.

The results of baropodometric measurements on the footplate “FootplatePro” from the German company “Currex”, Hamburg were used in order to classify the subjects into groups according to the feet status [21,22]. The “FootplatePro” is a sensor board comprising 4096 sensors distributed on an area of 490 × 490 mm, the frequency of which totals 200 Hz, with a capacitive sensor type and digital automatic calibration. The “FootplatePro” measures the load distribution on the soles in standing position and while walking. Thus, it can be used to measure both static and dynamic feet function. All instruments, devices, and systems in the sports laboratory have undergone strict calibration procedures and preparations for laboratory measurements.

For this work, the footprint results of the 30 s standing test on the sensor board were used. The subjects are positioned on a board with feet hip-width apart with equal distances of both feet from the longitudinal midline. The melleoli are at the height of the transverse midline. Thus, the view of the test subject is straight at eye level height towards the image of a dinosaur that serves to maintain the attention of a child. Before placing it on the board, each child is prepared in advance. The obtained baropodometric results of the footprint were evaluated by the three most experienced experts independently of each other. Out of the total number, there were disagreements concerning only 2 test subjects who were finally evaluated by a joint substantiated decision.

#### 2.3.4. Motor Status

Modified motor tests, modified according to Bala G, Stojanović MV, and Stojanović M [23], were utilised to assess preschool children’s motor status. A battery of 7 motor tests was applied. More precisely, in order to assess the movement structuring factors, the following tests were applied: (1) running at 20 m from a high start (RUN), the time being measured in tenths of a second; (2) standing broad jump (SBJ) from the stationary position, the result being measured in centimetres; (3) backwards polygon (BPL), the time being measured in tenths of a second, in order to assess the tone factors and synergistic regulation; (4) rectangular seated forward bend (SFB), the result being measured in centimetres; (5) plate tapping (PLT), the result being represented by means of the number of double touches of the hand on the marked squares at a distance of 40 cm from each other, achieved in a time of 15 s, in order to assess the intensity regulation factors and the duration of motor units’ excitation: (6) sit-ups for 60 s (SUP), the result being the total number of correctly performed torso lifts during the given time; and (7) bent arm hang (BAH), the result being measured in tenths during which the test subject performed the task in the correct manner. The tests were arranged in such a way as to avoid the influence of one test on another. Scorers on all tests have been conducting tests continuously since the school year 2007/2008, which, at that time, was the thirteenth time of performing scoring in a row and had been previously selected based on specific abilities for a particular test.

### 2.4. Statistical Data Processing

The statistical program (SPSS 26) was utilised for the purpose of data processing. Descriptive statistics were made. The statistical procedures of nonparametric statistics according to the Kruskal–Wallis method were used in order to examine the statistical significance of differences between groups of test subjects with different feet statuses in the manifestation of motor status. The Mann–Whitney U test was utilised to determine the size of the differences between the observed groups classified concerning the feet status in the manifestation of motor abilities, all being at the level of significance *p* ≤ 0.05.

## 3. Results

Table 1 shows the results: Out of a total of 202 respondents aged 3.9 to 6.5 years, 141 (69.8%) were male body height 117.3 ± 7.1 and body weight 22 ± 3.7 and 61 (30.2%) female respondent’s body height 114.9 ± 7.4 and body weight 21.2 ± 3.8.

With a normal arch of the foot, there were 30 (14.9%) subjects, 90 (44.6%) with a high arch of the foot, 41 (20.3%) with flat feet and 41 (20.3%) subjects who had different left and right foot status. There are 17 boys (12.1%) and 13 girls (21.3%) with a normal arch of the foot, and 32 (22.7%) boys and 9 (14.8%) girls with flat feet.

Table 2 shows the results: Undernourished were 6 (3%), overfed 30 (14.9%), obese 13 (6.4%), and normally fed 153 (75.7%). A higher percentage are of normally fed boys 110 (78%) while the girl was 43 (70%). Girls are more likely to be overweight 14 (23%), and less often, obese 2 (3.3%) while boys are more often obese 11 (7.8%), and less often with overweight 16 (11.3%).

Table 3 displays the results of statistical significance of group differences according to the feet status in the results of the applied battery of motor tests. A statistically significant difference was detected that existed between the groups in all tests except for the group of rectangular seated forward bend (SFB), with a level of significance *p* ≤ 0.05. Even for tests: BPL; PLT; SBJ; RUN the statistical significance of the differences is at level *p* ≥ 0.01. The difference was determined even for the backward polygon tests (BPL), plate tapping (PLT), and standing broad jump (SBJ). The statistically significant difference was detected at the level of *p* ≤ 0.01 for running at 20 m from a high start (RUN). By comparing the mean ranks, we concluded that the test subjects with high arched feet showed the best results in all tests, followed by the test subjects with normal arch feet.

Furthermore, by utilising the analysis of descriptive group statistics, defined by feet status, it was found that the test subjects with high arched feet were 0.2 decimal years older than the subjects with normal arch feet, the same as those older than subjects with flat arch feet, which means that the subjects with high arched feet were 0.4 years older than the subjects with flat feet, which can have a significant impact on better motor test results. This observation can also be confirmed by the higher average body height of the subjects with high arched feet (body height = 118 ± 6.9), normal arched feet (body height = 114.6 ± 7.8), and flat feet (body height 112.2 ± 6.1). Therefore, when concluding, it is necessary to consider these facts.

Table 4 shows the results of the Mann–Whitney U test for assessing the statistical significance of the difference between the groups with normal arch feet and high arched feet in the results of motor tests. This test also shows that the subjects with high arched feet showed better results in tests of motor skills compared to the subjects with a normal arch of the foot, but without statistically significant differences at the level of *p* ≤ 0.05. The effects size according to Cohen’s criterion for this test is small, with values from 0.064 to 0.196.

Table 5 displays the results of the Mann–Whitney U test, which show that there is no statistically significant difference in the manifestation of motor status between the groups of test subjects with flat arch and normal arch feet. However, the values of the middle ranks show that significantly better results in motor status are still achieved by the test subjects with a normal arch of the foot in all the conducted tests. The effects size according to Cohen’s criterion for this test is also small, with values from 0.096 to 0.235.

Table 6 displays the results of differences in the manifestation of motor status between the test subjects with high arched feet and flat arches of the feet. The results show that subjects with high arched feet show better motor status in all tests at the level of significance of *p* ≤ 0.01 except in the test rectangular seated forward bend (SFB) at the level of *p* ≤ 0.05. The effects size according to Cohen’s criterion for this test is medium for the tests backwards polygon, plate tapping, standing broad jump, and sit-ups for 60 s and small for the tests rectangular seated forward bend and bent arm hang.

## 4. Discussion

The results of the nutritional status of the test subjects on the total sample in this study are in accord with the results of other studies in which the percentage of the obese and overweight in the population of Serbian children ranges from 15 to 25% [24,25].

The research dealing with the relationship between motor skills and feet status in preschool children is rare or almost non-existent. The results of the previous research revealed that overweight and obesity in preschool children inhibit their physical activity and reduce the possibility of manifesting motor status [26]. In order to avoid the large negative impact of being overweight on the manifestation of motor status and the status of the arches of the feet, the final statistical processing included solely the data of the test subjects with normal nutrition [26,27,28,29,30].

The unexpectedly large number of test subjects with high arched feet could be explained by the fact that only subjects with normal nutrition were included in the data processing sample. On the other hand, perhaps the reason for this lies in the fact that the test subjects included in this study (ages 3.9 to 6.5 decimal years) have not yet completed the development of the arches of the feet, and a larger number of children with normal arch feet was not expected [8,9]. Additionally, at this age, there is still valgus of the heel and valgus in the posture that separates the outer and central arch of the foot, which makes the image of the footprint appear with a high arch, i.e., with contact surfaces on the heel and the front part of the sole [31,32]. When examining the relationship between the explosive power of the legs and the longitudinal arch of the feet of nine-year-old test subjects, it has been found that there is no statistically significant difference between the groups defined by feet status in motor tests [33,34].

In the sample of the test subjects investigated in our study, the explosive power of the legs or any other ability cannot be considered because there is still no differentiation of motor skills in children of this age. However, one can argue that some have better results in some motor tests or generally have better motor skills [14,15].

The results of this study reveal that there is a statistical significance of differences between the groups defined by the status of the feet even at a significance level of *p* ≥ 0.01 for the tests backwards polygon (BPL), plate tapping (PLT), standing broad jump (SBJ), and running at 20 m from a high start (RUN), and at the significance level of *p* ≤ 0.05, a statistically significant difference was found in the sit-ups for 60 s (SUP) and bent arm hang (BAH). It is only in the leaning rectangular seated forward bend (SFB) test that no statistical significance of the group differences has been found.

In order to determine the statistical significance of the difference between all groups, we tested the differences using the Mann–Whitney U test. A statistically significant difference in the manifestation of motor abilities between the test subjects with normal and flat feet has not been found. Therefore, the effects size according to Cohen’s criterion for this test is small. However, it is evident that the subjects with normal arch feet show significantly better results than subjects with flat soles. The test subjects with a high arch showed better results in all tests compared to the subjects with a normal arch, but not statistically significant at the significance level of *p* ≤ 0.05, and the effects size according to Cohen’s criterion for this test is small. Statistically, significantly better results in all tests at a level of significance *p* ≤ 0.01 were shown by the subjects with a high arch in relation to subjects with a flat arch, except in the test of rectangular seated forward bend (SFB), for which showed better results at the level of significance *p* ≤ 0.05. The effects size of Cohen’s criterion for this test is medium, except for the tests rectangular seated forward bend and bent arm hang, in which the effect size is small. The high arched feet can also be brought into relation with the beginning of the period of rapid growth and development in which the skeleton grows first—more precisely, the lower leg [35,36]—which leads to the separation of the outer, middle part of the sole, thereby creating an image of the high arch, which is the transitional phase in feet development. This fact, which should be considered, refers to the finding that the test subjects with high arched feet are the tallest and the oldest, which can further affect the results of the rectangular seated forward bend test (SFB), as well as all the other tests.

## 5. Conclusions

Feet status had a statistically significant effect on the results of motor tests in preschool children. The best test results were exhibited by the group with high arched feet, followed by those with normal arch feet. Considering that motor development is most tumultuous at this age and the fact that what is missed in this period is difficult to compensate for during future development, we conclude that it is essential to stimulate the function and development of arches in preschool children.

The reason for the slow development of the arches probably lies in hypokinesia and inadequate footwear or footwear in general, which does not allow the development of the muscle stabiliser of the ankle joint as well as the pelvic stabiliser. Therefore, it is desirable to create a program and conditions under which the program would be implemented in kindergarten institutions to encourage the development of foot function through athletic activities of walking, running, jumping, hopping, and polygons with balancers of different types.

In addition to this, it is also necessary for the surface to be hard and non-slippery and for the children to conduct the program in ballet shoes with a thin rubber, non-slippery sole in order to attenuate the impact during walking or landings with their foot and lower leg muscles in the course of the mentioned movement activities. Above all, the recommended activities would encompass exercises and balances on one leg through the positions of the scales or exercises on various types of balancers. Thus, over time, children will learn to run and jump properly in the right way. Additionally, this will encourage the formation of the arches of their feet, thereby enabling more efficient motor development.

## 6. The Limitations of the Study and Future Research Agenda

Feet status in children could have an important relationship on adulthood running gait or on possible lower-limb tendon injuries [37,38]. Based on this fact, a future research agenda should be developed.

## Figures and Tables

**Table 1:** Demographic data separated by gender.

Descriptive Statistics (Gender = 1)										
	**N**	**Minimum**	**Maximum**	**Mean**	**Std. Deviation**	**Variance**	**Skewness**		**Kurtosis**	
	**Statistic**	**Statistic**	**Statistic**	**Statistic**	**Statistic**	**Statistic**	**Statistic**	**Std. Error**	**Statistic**	**Std. Error**
age	141	3.9	6.5	5.321	0.7448	0.555	−0.184	0.204	−1.079	0.406
Height	141	102.1	138.0	117.305	7.0644	49.905	0.261	0.204	0.050	0.406
Weight	141	14.7	37.6	21.983	3.7544	14.095	1.098	0.204	2.683	0.406
BPL	141	105	583	222.01	80.397	6463.750	1.540	0.204	2.972	0.406
PLT	141	9	28	20.44	3.754	14.091	−0.331	0.204	−0.437	0.406
SFB	141	19	54	37.36	7.415	54.975	−0.007	0.204	−0.279	0.406
SBJ	141	0	149	108.50	23.735	563.352	−1.833	0.204	6.383	0.406
SUP	141	0	44	21.75	9.927	98.545	−0.373	0.204	−0.332	0.406
BAH	141	0	586	143.73	102.653	10,537.555	1.961	0.204	5.270	0.406
RUN	141	41	91	50.52	6.818	46.480	2.857	0.204	13.043	0.406
Valid N (listwise)	141									
**Descriptive Statistics (Gender = 2)**										
	**N**	**Minimum**	**Maximum**	**Mean**	**Std. Deviation**	**Variance**	**Skewness**		**Kurtosis**	
	**Statistic**	**Statistic**	**Statistic**	**Statistic**	**Statistic**	**Statistic**	**Statistic**	**Std. Error**	**Statistic**	**Std. Error**
age	61	3.9	6.5	5.136	0.7303	0.533	0.208	0.306	−1.049	0.604
Height	61	98.0	134.7	114.916	7.4258	55.143	0.209	0.306	−0.009	0.604
Weight	61	13.6	32.8	21.169	3.7595	14.134	0.667	0.306	0.652	0.604
BPL	61	106	496	269.79	96.326	9278.704	0.487	0.306	−0.667	0.604
PLT	61	13	26	19.05	3.528	12.448	0.191	0.306	−1.033	0.604
SFB	61	23	63	43.20	7.713	59.494	0.116	0.306	0.345	0.604
SBJ	61	60	144	101.43	19.803	392.149	0.191	0.306	−0.819	0.604
SUP	61	0	47	21.39	10.984	120.643	0.119	0.306	−0.469	0.604
BAH	61	0	680	144.02	134.763	18,161.050	2.089	0.306	5.118	0.604
RUN	61	42	66	53.13	6.394	40.883	0.269	0.306	−0.940	0.604
Valid N (listwise)	61									

Legend: BPL—backwards polygon, PLT—plate tapping, SFB—rectangular seated forward bend, SBJ—standing broad jump, SUP—sit-ups for 60 s, BAH—bent arm hang, RUN—running at 20 m from a high start; 1 is male gender, 2 is female gender.

**Table 2:** Nutrition of respondents by BMI.

		Frequency	Percent	Valid Percent	Cumulative Percent
Valid	undernourished	6	3.0	3.0	3.0
	normally	153	75.7	75.7	78.7
	overfed	30	14.9	14.9	93.6
	obese	13	6.4	6.4	100.0
	Total	202	100.0	100.0	

**Table 3:** The difference in the manifestation of motor status between the subjects with different feet status.

		BPL	PLT	SFB	SBJ	SUP	BAH	RUN
Feet Status	No.	Mean Rank	Mean Rank	Mean Rank	Mean Rank	Mean Rank	Mean Rank	Mean Rank
**Normal arch feet**	21	66.33	55.17	57.76	56.21	60.71	61.36	67.64
**High arched feet**	70	52.62	69.40	65.53	67.94	66.22	66.08	51.79
**Flat feet**	29	75.29	42.88	50.34	45.66	46.53	46.41	76.34
**Total**	120							
**Kruskal–Wallis H**		9.427	12.600	4.072	8.807	6.579	6.570	11.341
**Significant difference**		0.009 **	0.002 **	0.131	0.012 **	0.037 *	0.037 *	0.003 **

** The statistically significant difference at the level of *p* ≤ 0.01; * The statistically significant difference at the level of *p* ≤ 0.05. Legend: BPL—backwards polygon, PLT—plate tapping, SFB—rectangular seated forward bend, SBJ—standing broad jump, SUP—sit-ups for 60 s, BAH—bent arm hang, RUN—running at 20 m from a high start.

**Table 4:** The difference in the manifestation of motor status between the subjects with normal arch feet and high arched feet.

		BPL	PLT	SFB	SBJ	SUP	BAH	RUN
Feet Status	No.	Mean Rank	Mean Rank	Mean Rank	Mean Rank	Mean Rank	Mean Rank	Mean Rank
**Normal arch feet**	21	54.05	37.86	41.62	39.64	42.93	42.83	55.40
**High arched feet**	70	43.59	48.44	47.31	47.91	46.92	46.95	43.18
**Total**	91							
**Mann–Whitney U**		566.000	564.000	643.000	601.500	670.500	668.500	537.500
**Wilcoxon W**		3051.000	795.000	874.000	832.500	901.500	899.500	3022.500
**Z**		−1.592	−1.617	−0.867	−1.258	−0.608	−0.626	−1.866
**r**		0.167	0.169	0.091	0.132	0.064	0.066	0.196
**Significant difference**		0.111	0.106	0.386	0.208	0.543	0.531	0.062

Legend: BPL—backwards polygon, PLT—plate tapping, SFB—rectangular seated forward bend, SBJ—standing broad jump, SUP—sit-ups for 60 s, BAH—bent arm hang, RUN—running at 20 m from a high start, z—z-score (standard score) r—effect size.

**Table 5:** The difference in the manifestation of motor status between the subjects with normal arch feet and flat arch feet.

		BPL	PLT	SFB	SBJ	SUP	BAH	RUN
Feet Status	No.	Mean Rank	Mean Rank	Mean Rank	Mean Rank	Mean Rank	Mean Rank	Mean Rank
**Normal arch feet**	21	23.29	28.31	27.14	27.57	28.79	29.52	23.24
**Flat arch feet**	29	27.10	23.47	24.31	24.00	23.12	22.59	27.14
**Total**	50							
**Mann–Whitney U**		258.000	245.500	270.000	261.000	235.500	220.000	257.000
**Wilcoxon W**		489.000	680.500	705.000	696.000	670.500	655.000	488.000
**Z**		−0.914	−1.164	−0.679	−0.855	−1.358	−1.662	−0.936
**r**		0.130	0.165	0.096	0.121	0.192	0.235	0.132
**Significant difference**		0.361	0.244	0.497	0.392	0.174	0.097	0.349

Legend: BPL—backwards polygon, PLT—plate tapping, SFB—rectangular seated forward bend, SBJ—standing broad jump, SUP—sit-ups for 60 s, BAH—bent arm hang, RUN—running at 20 m from a high start, z—z-score (standard score) r—effect size.

**Table 6:** The difference in the manifestation of motor status between the subjects with high arched feet and flat arch feet.

		BPL	PLT	SFB	SBJ	SUP	BAH	RUN
Feet Status	No.	Mean Rank	Mean Rank	Mean Rank	Mean Rank	Mean Rank	Mean Rank	Mean Rank
**High arched feet**	70	44.54	56.46	53.71	55.53	54.80	54.63	44.11
**Flat arch feet**	29	63.19	34.41	41.03	36.66	38.41	38.83	64.21
**Total**	99							
**Mann–Whitney U**		632.500	563.000	755.000	628.000	679.000	691.000	603.000
**Wilcoxon W**		3117.500	998.000	1190.000	1063.000	1114.000	1126.000	3088.000
**Z**		−2.941	−3.488	−2.001	−2.977	−2.586	−2.491	−3.175
**r**		0.295	0.350	0.201	0.299	0.260	0.250	0.319
**Significant difference**		0.003 **	0.000 **	0.045 *	0.003 **	0.010 **	0.013 **	0.001 **

* The statistically significant difference at the level of *p* ≤ 0.05, ** The statistically significant difference at the level of *p* ≤ 0.01. Legend: BPL—backwards polygon, PLT—plate tapping, SFB—rectangular seated forward bend, SBJ—standing broad jump, SUP—sit-ups for 60 s, BAH—bent arm hang, RUN—running at 20 m from a high start, z—z-score (standard score), r—effect size.

## Data Availability

Data supporting the reported results can be found at the link: THE RELATIONSHIP BETWEEN FEET STATUS AND MOBILITY STATUS IN.xlsx—Google Drive Access date: 16 June 2021.

## References

[1] Al Abdulwahab S.S., Kachanathu S.J. (2015). The effect of various degrees of foot postures on standing balance in healthy adult population. Somatosens. Mot. Res..

[2] Jonely H., Brismée J.M., Sizer P.S., James C.R. (2011). Relationships between clinical measures of static foot posture and plantar pressure during static standing and walking. Clin. Biomech..

[3] Butković I. (2009). Povrede i Oboljenja Stopala i Skočnog Zgloba.

[4] Rodriguez N., Volpe R.G. (2010). Clinical diagnosis and assessment of the pediatric pes planovalgus deformity. Clin. Podiatr. Med. Surg..

[5] Müller S., Carlsohn A., Müller J., Baur H., Mayer F. (2012). Static and dynamic foot characteristics in children aged 1–13 years: A cross-sectional study. Gait Posture.

[6] Pfeiffer M., Kotz R., Ledl T., Hauser G., Sluga M. (2006). Prevalence of flat foot in preschool aged children. Pediatrics.

[7] Evans A., Nicholson H., Zakarias N. (2009). The paediatric flat foot proforma (p-FFP): Improved and abridged following a reproducibility study. J. Foot Ankle Res..

[8] Leung A.K.L., Cheng J.C.Y., Mak A.F.T.A. (2005). Cross-sectional study on the development of foot arch function of 2715 Chinese children. Prosthet. Orthot. Int..

[9] Evans A.M., Rome K.A. (2011). Cochrane review of the evidence for non-surgical interventions for flexible pediatric flat feet. Eur. J. Phys. Rehabil. Med..

[10] Razeghi M., Edward M.B. (2002). Foot type classification: A critical review of current methods. Gait Posture.

[11] Uden H., Scharfbillig R., Causby R. (2017). The typically developing paediatric foot: How flat should it be?. A systematic review. J. Foot Ankle Res..

[12] Banwell H.A., Paris M.E., Mackintosh S., Williams C.M. (2018). Paediatric flexible flat foot: How are we measuring it and are we getting it right?. A systematic review. J. Foot Ankle Res..

[13] Bala G., Kiš M., Popović B. (1996). Trening u razvoju motoričkog ponašanja male dece. Godišnjak.

[14] Ismail A.H., Gruber J.J. (1971). Integrated Development—Motor Aptitude and Intellectual Performance.

[15] Bala G. (1981). Struktura i Razvoj Morfoloških i Motoričkih Dimenzija dece SAP Vojvodine.

[16] Đorđić V., Uvod U., Bala G. (2006). Fizička Aktivnost Devojčica i Dečaka Predškolskog Uzrasta 7–12.

[17] World Health Organization (2009). WHO Child Growth Standards: Growth Velocity Based on Weight, Length and Head Circumference: Metods and Development.

[18] Cavanagh P.R., Rodgers M.M. (1987). The arch index: A useful measure from footprints. J. Biomech..

[19] Rosenbaum D., Muller B., Wolf C. (2016). Assessing pediatric foot deformities by pedobarography. Handbook of Human Motion.

[20] Lohman T.G., Roche A.F., Martorel R. (1988). Anthropometric Standardization Reference Manual.

[21] Behling A.-V., Manz S., von Tscharner V., Nigg B. (2020). Pronation or foot movement–what is important. J. Sci. Med. Sport.

[22] Hagen L., Pape J.P., Kostakev M., Peterlein C.-D. (2019). Pedobarographic changes during first month after subtalar extra-articular screw arthroereisis (SESA) operation of juvenile flexible flatfoot. Arch. Orthop. Trauma Surg..

[23] Bala G., Stojanović M.V., Stojanović M. (2007). Merenje i Definisanje Motoričkih Sposobnosti Dece.

[24] Despotović M., Aleksopulos H., Despotović M., Ilić B. (2013). Stanje uhranjenosti dece predškolskog uzrasta. Med. Časopis.

[25] Stupar D., Popović B., Vujović P. (2014). Stanje uhranjenosti predškolske dece Novog Sada. Glas. Antropološkog Društva Srb..

[26] Musalek M., Kokstejn J., Papez P., Scheffler C., Mumm R., Czernitzki A.F., Koziel S. (2017). Impact of normal weight obesity on fundamental motor skills in pre-school children aged 3 to 6 years. Anthropol. Anz..

[27] Castetbon K., Andreyeva T. (2012). Obesity and motor skills among 4 to 6-year-old children in the United States: Nationally-representative surveys. BMC Pediatr..

[28] Khalaj N., Amri S. (2014). Mastery of gross motor skills in preschool and early elementary school obese children. Early Child Dev. Care.

[29] Mickle K., Steele J.R., Munro B.J. (2006). The feet of overweight and obese young children: Are they flat or fat?. Obesity.

[30] Mueller S., Carlsohn A., Mueller J., Baur H., Mayer F. (2016). Influence of obesity on foot loading characteristics in gait for children aged 1 to 12 years. PLoS ONE.

[31] Mickle K., Steele J.R., Munro B.J. (2008). Is the foot structure of preschool children moderated by gender?. J. Pediatric Orthop..

[32] Chang H., Lin C., Kuo L., Tsai M.J., Chieh H.F., Su F.C. (2012). Three-dimensional measurement of foot arch in preschool children. Biomed. Eng. Online.

[33] Mihajlović I., Petrović M., Šolaja M. (2012). Differences in manifestation of explosive power of legs regarding to longitudinal foot arch in young athletes. Sport Mont.

[34] Aleksandrović M., Kottaras S. (2015). Does pes planus precondition diminish explosive leg strength: A pilot study. Facta Univ. Ser. Phys. Educ. Sport.

[35] Cameron N. (2012). Human Growth and Devolopment.

[36] Cunningham C., Scheuer L., Black S. (2016). Developmental Juvenile Osteology.

[37] Milic M., Erceg M., Palermi S., Iuliano E., Borrelli M., Cè E., Esposito F., Padulo J. (2020). Uphill walking at iso-efficiency speeds. Biol. Sport.

[38] Tarantino D., Palermi S., Sirico F., Corrado B. (2020). Achilles Tendon Rupture: Mechanisms of Injury, Principles of Rehabilitation and Return to Play. J. Funct. Morphol. Kinesiol..

